# Decoding the Neuroprotective Potential of Methyl Gallate-Loaded Starch Nanoparticles against Beta Amyloid-Induced Oxidative Stress-Mediated Apoptosis: An In Vitro Study

**DOI:** 10.3390/pharmaceutics13030299

**Published:** 2021-02-25

**Authors:** Nallasamy Prakashkumar, Bhagavathi Sundaram Sivamaruthi, Chaiyavat Chaiyasut, Natarajan Suganthy

**Affiliations:** 1 Department of Nanoscience and Technology, Alagappa University, Karaikudi 630003, India; prakashkumarnallasamy@gmail.com; 2 Innovation Center for Holistic Health, Nutraceuticals and Cosmeceuticals, Faculty of Pharmacy, Chiang Mai University, Chiang Mai 50200, Thailand; sivasgene@gmail.com

**Keywords:** Alzheimer’s disease, starch-encapsulated methyl gallate (SEMG), Neuro2A cells, β-amyloid peptide, oxidative stress, apoptosis

## Abstract

Alzheimer’s disease (AD) is a multifaceted neuronal disorder and a challenge to medical practitioners, as the blood–brain barrier (BBB) acts as a major obstacle for drug delivery to the brain. Development of a nanomaterial-based drug delivery system (DDS) paved a way to penetrate the BBB. Starch, a ubiquitous natural biopolymer, has received much attention as a DDS due to its biocompatibility, biodegradability and eco-friendly nature. The present study focuses on encapsulating methyl gallate (MG) within starch nanoparticles (starch-encapsulated MG (SEMG)) and assesses its neuroprotective potential against β-amyloid (Aβ)-induced toxicity, the key factor for AD pathogenesis in Neuro2A cells. SEMG showed potent acetylcholinesterase inhibitory, antioxidant activity and anti-amyloidogenic activity by attenuating the fibrillation of Aβ and destabilizing the preformed mature fibrils. Furthermore, SEMG also attenuated the cytotoxic effect induced by Aβ in Neuro2A cells (50% inhibitory concentration 18.25 ± 0.025 μg/mL) by mitigating reactive oxygen species (ROS)-mediated macromolecular damage, restoring mitochondrial membrane potential and attenuating apoptosis. Characterization of SEMG revealed amorphous rock-shaped structure with average particle size of 264.6 nm, exhibiting 83% loading efficiency and sustained release of drug, with 73% release within 24 h at physiological pH. Overall, the outcome of the present study signifies starch as a promising nanocarrier for the delivery of drugs for the treatment of AD.

## 1. Introduction

Starch is an inexpensive renewable polysaccharide ubiquitously found in nature with wide application in industries. Multiple evidences revealed the use of this biopolymer as an encapsulating agent to preserve compounds such as vitamins, lipids, drugs, microorganisms and flavoring agents, polyphenols and herbicides from adverse conditions such as light, temperature and pH, etc. [1]. Mesoporous microstructure of starch, diverse functionalization retaining its physicochemical characteristics and stimuli-based release of drugs reveals starch as a suitable encapsulating agent for delivery of drugs. Starch from natural sources such as potato, maize, corn and cassava, and nonconventional source like amaranth, taro and tapioca, were widely used for encapsulation processes [2,3]. Owing to its biocompatibility, microporosity and biodegradability, starch has been chosen for nanoencapsulation in the present study.

Alzheimer’s disease (AD) is a devastating brain-related degenerative disorder affecting mostly the elderly population above 65 years, and it is considered as one of the greatest medical challenges of the 21st century. The increase in the aging population globally increases the incidence of AD, and currently, around 46.8 million people are affected by dementia, which is supposed to double by 2030 and quadruple by 2050 if no effective therapy intervenes [4]. Clinical symptoms include difficulty in remembering conversation and depression in early days, followed by memory impairment, difficulty in communication, poor judgment, behavioral changes, confusion and disorientation, leading to loss of activities of daily life in later stages, and ultimately leading to death [5]. Cardinal pathological hallmarks are deposition of β-amyloid (Aβ) plaques in the synaptic region and neurofibrillary tangles composed of hyperphosphorylated tau in the axonal region, which induces oxidative stress and inflammation-mediated degeneration of neurons primarily in the hippocampal region linked to learning and memory. Degeneration of neurons leads to imbalance in neurotransmitters like acetylcholine, dopamine and serotonin, affecting synaptic neurotransmission and leading to cognitive deficiencies [6]. Despite several researches, pathogenesis and effective treatment for AD have not yet been elucidated. FDA (Food and Drug Administration, United States)-approved drugs such as AChE (Acetylcholinesterase) inhibitors like donepezil, rivastigmine, galanthamine and N-methyl-d-aspartate (NMDA) receptor antagonist memantine have been used for the treatment of mild to moderate AD, however these drugs possess severe side effects [7]. Elucidation of the molecular mechanism of AD illustrated that AD is a multifactorial disorder, involving intertwined complex biochemical pathways, so hitting a single target will not be efficient for AD. Although several synthetic drugs were obtained by incorporating one or more pharmacophores within one scaffold, naturally occurring compounds with multipotent activity received much attention. Among the risk factors, aggregation of Aβ peptide plays a pivotal role in AD pathogenesis, so the current therapeutic strategy also focused on either blocking the amyloid aggregation and/or destroying the already formed aggregates. In silico, in vitro and in vivo studies revealed that several phenolic compounds such as curcumin, dopamine, derivatives of catechin, quercetin and rosmarinic acid showed multitargeted ability for AD therapeutics [8]. A recent report revealed that physical exercise together with intake of a diet rich in antioxidants, such as the Mediterranean diet, like olive oil, wine, fruits and vegetables rich in polyphenolic content and vitamins A, C and E, lowers the risk of AD by attenuating oxidative stress and inflammatory pathways involved in AD pathogenesis [9,10]. Another major limitation of AD therapy is the blood–brain barrier (BBB) and drug efflux by P- glycoprotein, which restricts the entry of drugs to the central nervous system (CNS). Nanotechnology-based approaches addressed this problem by adhering or entrapping/encapsulating AD drugs within nanocarriers, thereby promoting sustained release of entrapped drugs [11]. The proposed drug methyl gallate (MG) is a strong phenolic antioxidant widely found in *Terminalia myriocarpa, Terminalia chebula, Terminalia arjuna Bergenia ciliata (hairy Bergenia) Geranium niveum* and *Paeonia anomala* [12]. Methyl gallate has been reported for its wide pharmacological activities, such as antiplatelet action, antioxidant property, antiapoptotic, anticancer, antidiabetic and anti-inflammatory activities under in vitro and in vivo conditions [13,14]. Although methyl gallate has been reported for its biocompatible nature and neuroprotective effect via antioxidant, anticholinesterase and anti-aggregation properties [15], poor solubility, stability and inability to cross the BBB have turned into major obstacles in the usage of the drug for AD therapy. Hence, the present study focused on nano-encapsulating methyl gallate within a starch matrix to enhance its solubility, bioavailability and BBB permeability as a suitable candidate for AD therapy.

## 2. Materials and Methods

### 2.1. Reagents Required

Maize starch (C_6_H_10_O_5_)n, potassium ferricyanide, ferric chloride, sodium hydroxide (NaOH), cell culture medium DMEM (Gibco Dulbecco’s modified eagle medium), FBS (fetal bovine serum) and Trypsin EDTA (Ethylenediaminetetraacetic acid) were obtained from HiMedia laboratories Pvt Ltd., Mumbai India. Methyl gallate, DCFDH_2_DA (2′,7′-Dichlorofluorescin Diacetate), JC-1 (5,5′,6,6′-tetrachloro-1,1′,3,3′-tetraethylbenzimi-dazolylcarbocyanine iodide) and DPPH (2, 2-Diphenyl-1-Picrylhydrazyl) were obtained from Sigma Aldrich Pvt Ltd., MumbaiIndia.

### 2.2. Loading of Methyl Gallate onto Starch Nanoparticles

Methyl gallate was loaded into starch nanoparticles by the graft copolymerization method, in which MG was encapsulated within gelatinized starch [16]. About 5 g of maize starch was boiled in 0.4 M NaOH solution at 80 °C for gelatinization, followed by addition of 0.3% MG and acetone dropwise under constant stirring for 10 h at 60 °C. Reaction mixture was frequently extracted with acetone to remove the free MG. Starch-encapsulated MG (SEMG) was separated by centrifugation and dried at room temperature. Dry sample was ground to fine powder, followed by drying in a hot air oven at 80 °C for 12 h, and samples were stored in an airtight container at room temperature.

#### 2.2.1. Loading Efficiency of MG

Methyl gallate-loaded starch nanoparticles were assessed by quantifying the concentration of MG in supernatant at 270 nm using a UV–Visible spectrophotometer. The concentration of MG was calculated by a calibration graph using different concentrations of MG (10–50 μg/mL). Experiments were carried out in triplicates and the loading efficiency of MG (LE) was calculated as per the formula:

(1) $$\mathrm{Loading}\;\mathrm{efficiency}\;=\frac{{\left[\mathrm{MG}\right]}_{T}-\;{\left[\mathrm{MG}\right]}_{S}}{{\left[\mathrm{MG}\right]}_{T}}\;\times \;100$$

[MG]_T_—total amount of methyl gallate used for encapsulation, and [MG]s—methyl gallate in the supernatant.

(2) $$\begin{array}{l} \mathrm{Yield}\;\mathrm{Percentage}\;=\frac{\mathrm{Total}\;\mathrm{wt}\;\mathrm{of}\;\mathrm{Nanoparticles}}{\mathrm{Total}\;\mathrm{amount}\;\mathrm{of}\;\mathrm{MG}\;+\;\mathrm{Amount}\;\mathrm{of}\;\mathrm{starch}\;\mathrm{used}}\times 100 \end{array}$$

#### 2.2.2. Methyl Gallate Release Kinetics

Release kinetics of MG were assessed under in vitro condition based on the methodology of Winarti et al. [17]. Approximately 50 mg of MG was dissolved in 15 mL of phosphate buffered saline, pH 7.4, and subjected to constant stirring at 37 °C. At specific ntervals (4 h intervals for 24 h), the supernatant was collected and replaced with the same volume of fresh PBS (Phosphate buffered saline) solution. The amount of MG released in the supernatant was quantified by a UV–Visible spectrophotometer at 270 nm using a calibration graph with various doses of standard MG. Percentage of MG released at specific time intervals was calculated using the equation:

(3) $$\mathrm{Release}\;\mathrm{of}\;\mathrm{MG}\;\left(\%\right)\;=\;\frac{[\mathrm{Methyl}\;\mathrm{gallate}]{\;}_{\mathrm{Ref}}}{[\mathrm{Methyl}\;\mathrm{gallate}]{\;}_{\mathrm{loaded}}}\;\times \;100$$

#### 2.2.3. Swelling Studies

The swelling nature of MG-loaded starch nanoparticle was evaluated by suspending pre-weighted dried MG-loaded starch nanoparticles in 10 mL of phosphate-buffered saline (pH 7.4) at 37 °C. Weight of the swollen starch nanoparticle was determined at various time intervals. The swelling ratio was calculated based on the following equation:

(4) $$\mathrm{Swelling}\;\mathrm{Ratio}\;=\;\frac{\mathrm{Weight}\;\mathrm{of}\;\mathrm{wet}\;\mathrm{MG}\;-\;\mathrm{Weight}\;\mathrm{of}\;\mathrm{dry}\;\mathrm{MG}}{\mathrm{Weight}\;\mathrm{of}\;\mathrm{dry}\;\mathrm{MG}}$$

### 2.3. Characterization

Absorption spectrum of SEMG was assessed using a UV-Visible spectrophotometer (UV 2450, Shimadzu, Kanagawa, Japan) by scanning between 200 and 800 nm with resolution of 1 nm. Crystalline nature of SEMG and MG alone was characterized by X-ray diffraction using the X’ Pert PRO Analytical X-ray diffractometer with Cu Kα radiation, operating at a voltage of 40 kV and current of 30 mA (PANalytical, Etten Leur, Netherlands). FTIR (Fourier-transform infrared spectroscopy) spectra of SEMG and MG alone were analyzed using the FTIR-Nicolet Thermo spectrophotometer iS5 (USA) instrument by scanning between 4000 and 400 cm^−1^ with a resolution of 4 cm^−1^. Morphology of SEMG was examined using atomic force microscopy (AFM) using NT-MDT (NTEGRA modular Scanning Probe Microscopy. Particle size and charge of fabricated SEMG was evaluated using dynamic light scattering (DLS) and zeta potential (ZP) analyzer (Zetasizer Ver. 6.20, UK).

### 2.4. Assessment of Neuroprotective Potential of SEMG

#### 2.4.1. In Vitro Antioxidant Assay

##### Free Radical Scavenging Assay

Free radical scavenging activity of SEMG and MG was assessed by the DPPH scavenging assay based on the methodology of Shimada et al. [18]. Different doses of SEMG (10–50 µg/mL) were treated with 0.1 mM DPPH in a 96-well microtiter plate and incubated in the dark at RT (Reaction time) for 1 h at 37 °C, and the absorbance was measured at 517 nm using a multilabel reader (Molecular Device Spectramax M3, Softmax Pro V5 5.4.1 software. The percentage inhibition of DPPH radical was calculated.

##### Assessment of Total Reducing Capacity of SEMG

Reducing power of SEMG was evaluated based on the methodology of Oyaizu [19]. SEMG in different doses (10–50 µg/mL) in 0.2 M of phosphate buffer (pH 6.6) was treated with 1% potassium ferricyanide and incubated for 20 min, followed by treating with 10% Trichloroacetic acid (TCA). Reaction mixture was centrifuged at 650 rpm for 10 min. Supernatant was mixed with equal volume of water followed by addition of 0.1% FeCl_3_, and the absorbance was measured at 700 nm.

#### 2.4.2. Evaluation of Acetylcholinesterase Inhibitory Activity of SEMG

Acetylcholinesterase (AChE) inhibitory activity was assessed based on the methodology of Ingkaninan et al. [20], with slight modification. Various doses of SEMG (10–50 µg/mL) were treated with AChE (10 U/mL) in 0.1 M Tris-HCl buffer (pH 8.0), for about 2 h at RT, followed by the addition of 3 mM DTNB ((5,5′-dithiobis-(2-nitrobenzoic acid)). Enzyme reaction was initiated by the addition of ATCI (15 mM) and the absorbance was read at 405 nm every 30 s for 3 min using a multilabel reader (Molecular Device Spectramax M3, Softmax Pro V5 5.4.1 software). The percentage of inhibition was calculated as the percentage of rate of reaction of test compound in comparison with vehicle control:

(5) $$\%\mathrm{of}\;\mathrm{inhibition}\;=\;\frac{\mathrm{Specific}\;\mathrm{activity}\;\mathrm{of}\;\mathrm{control}\;-\;\mathrm{Specific}\;\mathrm{activity}\;\mathrm{of}\;\mathrm{treated}}{\mathrm{Specific}\;\mathrm{activity}\;\mathrm{of}\;\mathrm{control}}\;\times 100$$

#### 2.4.3. Assessment of Anti-Aggregation and Disaggregation Potential of SEMG

##### Preparation of Aβ Peptide Monomer

Aβ (25–35), purchased as lyophilized powder, was dissolved in 1,1,1,3,3,3,hexafluoro 2 propanol (HFIP), sonicated for 2 h. Reaction mixture was lyophilized, reconstituted in 1 mL milli-Q water to a final concentration of 1 mM and stored at –20 °C until further use.

##### Aβ Aggregation Kinetic Study

Kinetic study on effect of SEMG against Aβ aggregation was evaluated by treating freshly prepared solution of Aβ (25–35) monomers (100 μM) in Tris-HCl (pH 7.4) in the presence and absence of various doses of SEMG (25 and 50 μg/mL) for 96 h at 37°C. Every 6 h, aliquots of reaction mixture were taken and assessed for aggregation based on the Thioflavin-T (Th-T) assay.

##### Effect of SEMG on Aβ Aggregation

Freshly prepared solution of Aβ (25–35) monomers in Tris-HCl (pH 7.4) to final concentration of 100 µM was incubated at 37 °C for 20 h to form oligomers. SEMG (50 µg/mL) was incubated with oligomeric mixture for 48 h and aliquots were drawn from the incubation mixture at 20 and 48 h respectively, for spectrofluorimetric and confocal microscopy analysis by Th-T assay based on the methodology of Khurana et al. [21]. Aliquots of reaction mixture was treated with 5 μM Th-T in Glycine-NaOH buffer, pH 8.5, and the fluorescence intensity was measured at excitation/emission wavelength 450/485 nm in a multi-mode plate reader (Molecular Device Spectramax M3). For CLSM (Confocal Laser Scanning Microscopy) analysis, aliquots of Aβ (25–35) peptide sample were diluted 2 times with 5 μM Th-T and transferred onto a slide. Fluorescent signals were then visualized by the confocal laser microscope system (CLSM 710, Carl Zeiss, Germany) and processed by software (Zen 2011). The fluorescence intensity was visualized in each of three random fields of the sample.

##### Fibril Disaggregation Assay

Mature fibrils were formed by incubating Aβ (25–35) in Tris-HCl (pH 7.4) for 96 h. Ability of SEMG to destabilize the mature fibrils was assessed by incubating SEMG (50 μg/mL) for 9 days and the aliquots were drawn for spectroscopic and confocal microscopic studies.

#### 2.4.4. Assessment of Neuroprotective Effect of SEMG Against Aβ (25–35)-Induced Toxicity in Neuro2A Cells

Neuro2A cells obtained from the National Center for Cell Sciences (NCCS), Pune, India, were used as an in vitro model system to assess the β-amyloid (Aβ)-induced toxicity. Cells were cultured in DMEM medium containing 10% FBS and 1× penicillin-streptomycin in a CO_2_ incubator at 37 °C.

##### 3-(4,5-dimethylthiazol-2-yl)-2,5-diphenyl-2H-tetrazolium bromide (MTT) Assay

Pre-seeded Neuro2A cells (2 × 10^5^ cells/mL) were treated with various doses of Aβ (25–35) (10–50 µM) for 24 h followed by PBS wash and treatment with MTT (1 mg/mL) for 3 h at 37 °C. The cells were subjected to PBS wash and the blue-colored formazan crystals were solubilized by DMSO (Dimethyl sulfoxide). Absorbance was measured at 540 nm using a multi-plate reader. Protective effect of SEMG (20–100 μg/mL) was assessed by pre-treating with SEMG for 2 h prior to Aβ (25–35) at 20 μM concentration and incubated for 24 h, and then subjected to the MTT assay [22].

##### Measurement of Intracellular ROS Formation

Intracellular reactive oxygen species (ROS) level was evaluated by measuring the change in fluorescence as a result of oxidation of the fluorescent probe DCFH-DA (2ʹ,7ʹ-Dichlorofluorescin Diacetate) [23]. Neuro2A cells were pretreated with SEMG at its 50% inhibitory concentration (IC_50)_ (18.25 ± 0.025 µg/mL) for 2 h followed by Aβ (20 µM) for 24 h. Cells were harvested and treated with DCFH-DA (10 µM) for 30 min at 37 °C in the dark followed by PBS wash to remove the extracellular DCFH-DA, and examined under a fluorescent microscope (Nikon ECLIPSE, Ti-E, Japan). Quantification of intracellular ROS was carried out by incubating the cells in lysis buffer (10 mM Tris, 20 mM EDTA and 0.25% Triton X-100) and the intensity of fluorescence was measured using a multi-mode plate reader with the excitation/emission wavelength of 485/535 nm, respectively.

##### Evaluation of Mitochondrial Membrane Potential (ΔΨm)

Loss of ΔΨm in cells treated with SEMG was analyzed using lipophilic cationic dye (JC-1), based on the methodology of Sivandzade et al. [24]. Cell line was treated with SEMG for 24 h followed by treatment with JC-1 (1 µg/mL) for 30 min at 37 °C in the dark. After the incubation period, the excess dye was removed by PBS wash and the fluorescence intensity was measured at excitation/emission wavelength (488/525 nm) using a multi-mode plate reader. For morphological analysis, the cells were grown in cover slip and subjected to JC-1 staining, followed by visualization using fluorescence microscopy (Nikon ECLIPSE, Ti-E, Japan).

##### Assessment of Macromolecular Damage

Lipid peroxidation was assessed according to the method of Ohkawa et al. [25] and the results were expressed as mM of TBARS (Thiobarbituric acid reactive substance)/mg of protein, with MDA (Malondialdehyde) as standard. Protein carbonyl content was measured according to the method of Levine et al. [26] and the results are expressed as mM of free carbonyl content/mg of protein.

##### Morphological Assessment of Apoptosis

Acridine orange and ethidium bromide (AO/EtBr) dual-staining technique was used to visualize nuclear changes and apoptotic body formation of the characteristic features of apoptosis [27]. Following Aβ treatment, Neuro2A was washed with PBS and stained with AO/EtBr solution (1:1, *v/v*) to a final concentration of 10 µg/mL for 10 min. The cells were visualized under a fluorescence microscope (excitation and emission 490/525 nm). Each field of cells was photographed (magnification ×400) for calculation of the relative fluorescence intensity. The cells with condensed or fragmented nuclei were counted as apoptotic cells. All experiments were repeated thrice and about 100 stained cells were counted in 10 randomly selected fields.

### 2.5. Statistical Analysis

All the experiments were conducted in triplicate and one-way analysis of variance (ANOVA) (SPSS 17) was used to compare the mean values of each treatment. Significant differences between the means of parameters were determined by using the Duncan’s test (*p* < 0.05) comparing between the groups control vs treated. IC_50_ values were calculated using Probit software (Probit Software LTD, New York, NY, USA).

## 3. Results and Discussion

### 3.1. Nanoencapsulation of 7-Methyl Gallate

Loading of MG into starch nanoparticles was achieved through bonding behavior of sodium alkoxide ion pairs present in the gelatinized starch with the hydroxyl end of MG. The retro gradation kinetics degrades the crystalline nature of the starch by dislocating the units of amylose and amylopectin in starch due to the transfer of moisture content along with acetone during the process of drying. During the process of drying at 80 °C, the nano-encapsulated MG gets grafted as nanoparticles, i.e., SEMG. The observation of drug loading determines that about 87% of methyl gallate was encapsulated from the total amount of methyl gallate taken for nanoencapsulation. However, the percentage of yield for nano-encapsulated methyl gallate is around 88.3% from the 2:10 ratio of starch and methyl gallate.

#### 3.1.1. UV–Visible Spectral Analysis

**Figure 1:**
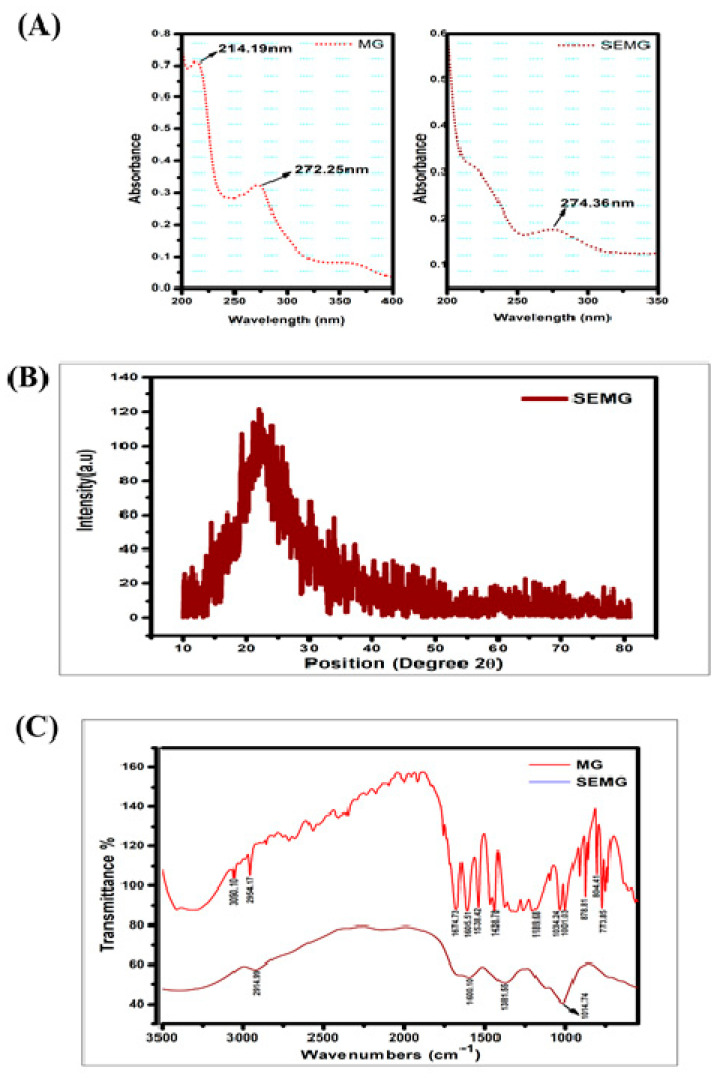
(**A**) UV-Visible spectrum of Methyl Gallate (MG) and Starch encapsulated methyl gallate (SEMG), (**B**) Powdered XRD spectrum of SEMG, (**C**) FTIR spectrum of SEMG (blue) and MG alone (red).

UV-Visible absorption spectra of MG showed peaks at 214 and 271 nm, in accordance with the report of Zhang et al. [28]. SEMG showed an absorption peak at 274 nm (Figure 1A), close to the MG spectrum. The slight change in SEMG peak is due to red shift corresponding to the removal of proton from the phenolic group of MG due to the addition of NaOH into MG during the process of nanoencapsulation. The bathochromic shift on SEMG is attributed to the fact that phenolic content of methyl gallate and starch nanoparticles were interconnected with hydrogen bonding [29].

#### 3.1.2. X-ray Diffraction (XRD) Pattern of SEMG

According to our previous investigation, nanoencapsulation of organic compounds such as drugs, enzymes or the ayurvedic compounds with starch exhibits amorphous nature [16]. In the present study, the crystalline nature of retrograded starch was altered due to the phase transfer on amylose and amylopectin units as a chain of polymer. Crystalline nature of MG was denatured due to the displacement of H_2_O with acetone from the gelatinized starch associated with MG during the process of drying [30], resulting in the formation of amorphous SEMG nanoparticles (Figure 1B).

#### 3.1.3. FTIR Spectra

FTIR spectra of MG alone and MG encapsulated with starch nanoparticles (SEMG) were shown in Figure 1C. Spectral variations of FTIR analysis were intensively noticed between MG and SEMG to find out the interaction of MG with starch nanoparticles. The major peaks for MG alone were observed at 3050.10, 2954.17, 1674.73, 1605.51, 1538.42, 1438.78, 1189.68, 1034.24, 1001.03, 878.81, 804.41 and 773.85 cm^–1^, corresponding to C–H stretch of aromatics, C=O stretch of general carbonyl group, C–C stretch in aromatic ring, N–O asymmetric stretching frequency of nitro compounds, C–N stretching frequency of aliphatic amines, C–O stretching frequency of esters, =C–H bends of alkenes and overtones of C-H bends. In case of SEMG, the peak intensities were probably altered in MG at 2914.99 cm^–1^, from 2954.17 cm^–1^ of alkanes C–H stretch, and 1601.10 cm^–1^, from 1605.51 cm^–1^ of C–C stretch in aromatic ring. The specific frequencies of esters at around 1000 cm^–1^ were observed in both MG and SEMG, specifying the ester derivative of gallic acid, i.e., methyl gallate [31]. On the other hand, the peak intensity was found at around 1381.55 cm^–1^ owing to the functional frequency for C–H bending of starch nanoparticles [32]. However, the significant spectral variation of FTIR for both MG and SEMG confirms the encapsulation kinetics in-between starch nanoparticles and MG.

#### 3.1.4. Morphology, Size and Stability of SEMG

**Figure 2:**
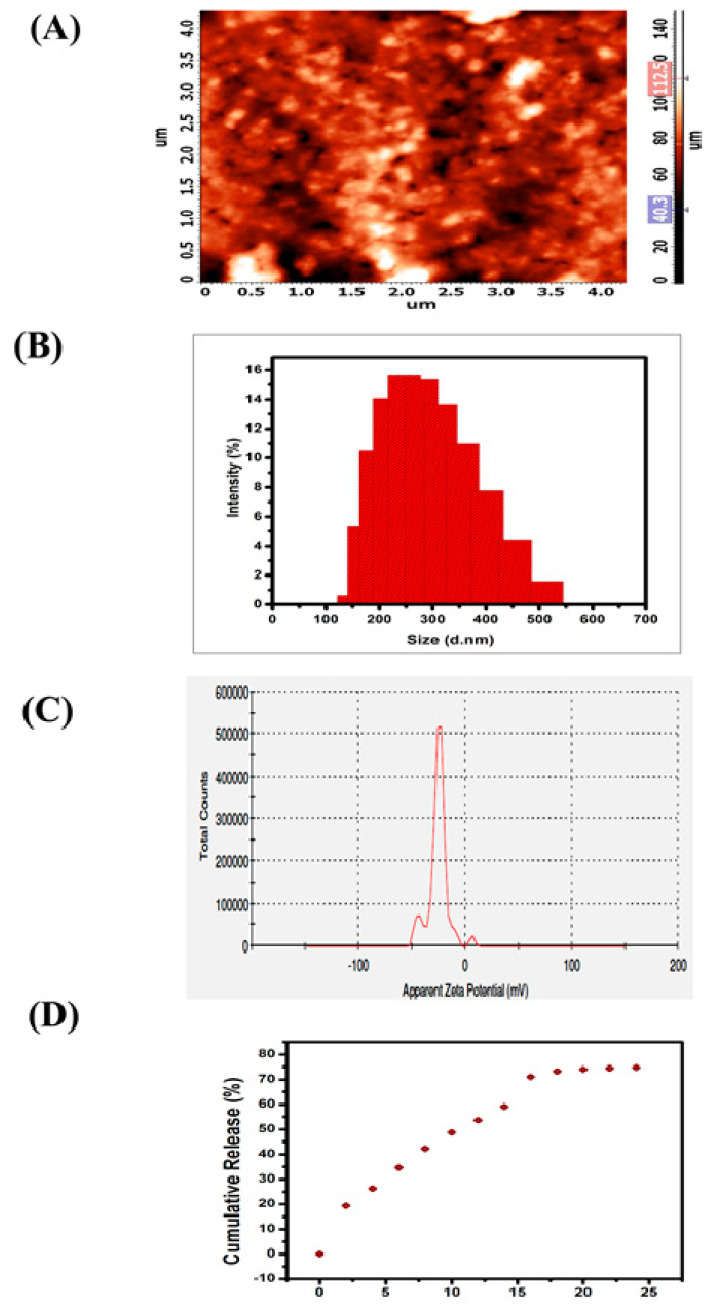
(**A**) Atomic Force Micrographs (AFM) of SEMG (2D and 3D image), (**B**) particle size distribution of the SEMG, (**C**) zeta potential measurement of SEMG, (**D**) In vitro release kinetics of Methyl Gallate (MG) from SEMG.

The two-dimensional (2D) micrograph of AFM displays the randomly arranged nanoparticles of SEMG without any kind of agglomeration, and the three-dimensional (3D) topography of AFM shows the possible protrudes of SEMG (Figure 2A). Morphology of SEMG revealed the presence of individual rock-shaped nanoparticles and the shapes of the particles were slightly uneven when compared to one another due to their amorphous nature, as revealed by XRD analysis.

DLS analysis revealed that the average particle size of SEMG was observed to be 264.6 nm (Figure 2B) based on the detection on Brownian motion of particles in fluids, and it was found to be significantly smaller than the particles of bulk starch, which was reported to be around 1000 nm and above [33]. Polydispersity index (PDI) value of SEMG is 0.170, which indicates middle range of particle dispersity due to the encapsulation kinetics between the starch nanoparticles and MG, i.e., graft co-polymerization. Kumari and Yadav [34] reported that cellular uptake of nanoparticles is possible up to a particle size of 500 nm via endocytosis. Based on this context, the cellular uptake of SEMG is possible as its size is less than 300 nm. Zeta potential analysis of SEMG was observed to be –25.4 mV (Figure 2C), and this kind of net negative charge nano-carrier facilitates the enhanced cellular uptake with no particle agglomeration through the electrostatic repulsion.

#### 3.1.5. In Vitro Drug Release Kinetics

In vitro drug release kinetics of SEMG were assessed in PBS (phosphate-buffered saline) at physiological pH 7.4 for 24 h under constant stirring. The surface erosion and swelling behavior of nano-carrier (SEMG) upon the PBS system initiates the process of breaking of hydrogen bonds between the starch nanoparticles and MG, which is significantly responsible for the release of MG [35]. Concentration of drug release was assessed by UV–visible spectroscopic analysis of supernatant collected every 4 h. The drug release of MG consecutively increased by ~12% ± 2% up to 16 h and the constant cumulative release was observed to be 73% during the period of 20 to 24 h, revealing the fact that maximum release of methyl gallate from the methyl gallate loaded starch nanocarrier (83%) was observed within 24 h (Figure 2D). Moreover, the swelling ratio was observed to be 3.9% in 24 h, when the drug release was observed to be 73%. This result illustrates SEMG as a suitable nano-carrier for the sustained release of MG.

#### 3.1.6. Neuroprotective Potential of SEMG

Alzheimer’s disease is the acquired dementia characterized by the progressive degeneration of neurons, leading to cognitive deficits and behavioral symptoms, which intensify with time, creating a major health challenge for the medical practitioners [36]. Current research on biomarkers of AD revealed that deposition of Aβ is the foremost and key pathogenic event in AD pathogenesis, which in turn promotes NFT formation, activating oxidative stress and inflammatory pathways, and ultimately leading to neuronal death. Failure in current therapeutic strategies for AD therapy is due to the blood–brain barrier (BBB), which blocks the entry of drugs into the central nervous system (CNS) [37]. Contemporary research revealed that fabricating nanomaterials within the nano platform enhances the efficiency of drugs through targeted drug delivery and sustained drug release, thereby minimizing side effects [38]. Although methyl gallate has been reported for its antioxidant and neuroprotective effects under in vitro conditions, poor solubility and gastrointestinal disturbances at higher doses limits its clinical efficiency. Hence, the present work focused on nano-encapsulating methyl gallate within starch biopolymer and evaluating its neuroprotective potential against Aβ-induced toxicity under in vitro conditions.

##### In Vitro Acetyl Cholinesterase Inhibitory and Antioxidant Activity

**Figure 3:**
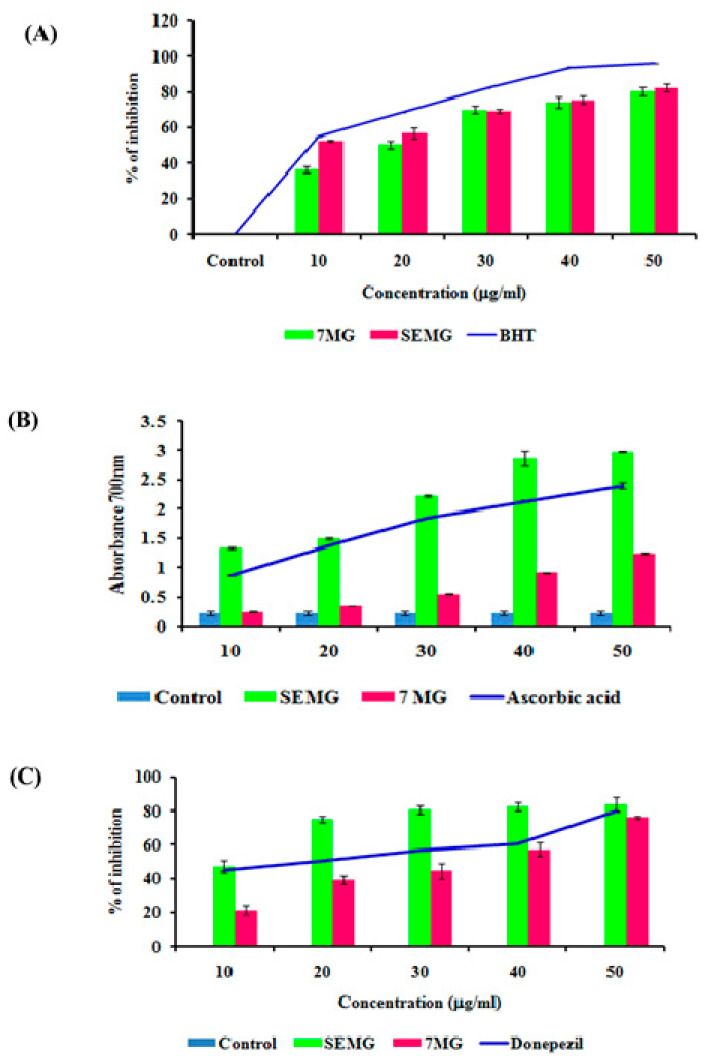
(**A**) Free radical scavenging activity of SEMG compared to standard Butylated hydroxytoluene, (**B**) Reducing power of SEMG in comparison with positive control ascorbic acid, (**C**) Acetylcholinesterase inhibitory activity of SEMG compared to standard drug donepezil. Results are expressed as Mean ± SD of triplicate assays.

In AD, the major causative agent for cognitive decline is degeneration of cholinergic neurons associated with loss of neurotransmission due to reduction in the level of neurotransmitter acetyl choline (ACh) [39]. As current therapeutic strategies focus on the use of acetylcholinesterase (AChE) inhibitors which restore the acetylcholine level, enhancing the cognitive function, the present study was carried out to assess the AChE inhibitory activity of SEMG. Results revealed that SEMG showed dose-dependent AChE inhibitory activity, with the highest inhibition rate of 83.75 ± 2.36 µg/mL at 50 µg/mL (Figure 3C), similar to the inhibitory effect of the same dose of MG and positive control (76% ± 1.40% and 80.05% ± 0.023%, respectively). IC_50_ value of SEMG and MG was observed to be 15.25 ± 0.024 and 35.25 ± 0.04 µg/mL, respectively. Results illustrate that starch encapsulation restored the AChE activity of MG, indicating it as a suitable drug delivery system.

Mounting evidences revealed that oxidative stress observed in early stages of AD plays a vital role in triggering complex signaling pathways, leading to pathogenesis of AD and lesion formation [40]. Antioxidant therapies were recently observed to be successful in preclinical trials for the treatment of AD. In the present study, the antioxidant capacity of SEMG was assessed and the results were shown in Figure 3A,B. Results of the present study illustrated that nano-encapsulated drugs showed potent free radical scavenging activity in a dose-dependent manner (10–50 μg/mL), with highest DPPH radical scavenging activity of 82.28% ± 2.17% at 50 μg/mL, similar to the same dose of standard BHT (Butylated hydroxytoluene) (96% ± 0.001%). IC_50_ values of nano-encapsulated drug, drug alone and standard BHT were observed to be 9.83 ± 0.05, 26.05 ± 0.025 and 12.04 ± 0.04 μg/mL, respectively.

Reducing capacity of compound depends on the electron-donating ability, which was assessed based on the ability to convert ferricyanide complex to ferrous form, and the change in color was measured at 700 nm. Figure 3A illustrated that SEMG showed increased absorbance (2.98 ± 0.008 a.u. (Arbitrary unit)) similar to positive control ascorbic acid (2.40 ± 0.05 a.u.) in a concentration-dependent manner, and higher than drug alone (1.24 ± 0.013 a.u.). Enhanced reducing ability of SEMG might be due to the synergistic effect of the antioxidant ability of starch nanoparticles and methyl gallate [15]. Results of the present study illustrate that nanoencapsulation of 7-MG retains the antioxidant capacity of the drug, revealing it as a suitable DDS.

##### Anti-Aggregation and Disaggregation Ability of SEMG

**Figure 4:**
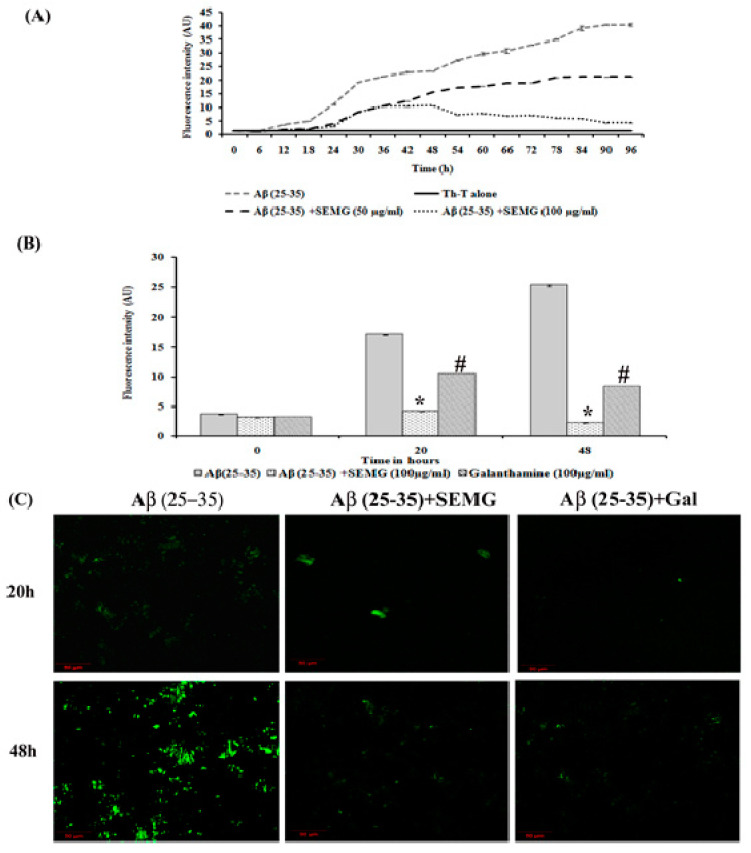
Anti-aggregation potential of SEMG against Aβ (25–35) preformed oligomers. (**A**) Sigmoidal curve representing aggregation kinetics of Aβ (25–35) from monomers to mature fibrils in the presence and absence of SEMG. (**B**) Th-T fluorescence assay. (**C**) Confocal microscopic analysis with 20 × magnification *,# *p* < 0.05 represents statistically significant difference in comparison with treated groups with control and positive control group.

Aberrant misfolding and aggregation of Aβ peptide into fibrils is the critical event which aggravates the pathogenesis of AD, hence researchers focused on investigating compounds that avert aggregation of Aβ peptide. Several phyto-compounds rich in polyphenolic content have been reported to attenuate the aggregation of Aβ peptide or destabilize the preformed fibrils. In addition, the drug MG has been reported for its potent anti-aggregation and Aβ mature fibril destabilizing ability [15]. The present study was carried out to assess whether MG retains the anti-aggregation ability after encapsulation within the starch nanoparticles. Thioflavin-T, a classical benzothiazole dye on intercalation between Aβ fibrils, exhibits enhanced fluorescent intensity, and this dye has been widely used as a probe for the detection of Aβ fibrillization process. Results of kinetics studies of Aβ fibrillation were shown in Figure 4A, which exhibited a sigmoidal curve revealing a time-dependent increase in fluorescence intensity, illustrating the polymerization of Aβ (25–35) monomers to fibrils at 48 h and mature plaques (96 h) when compared to Th-T blank alone.

The SEMG (50 and 100 μg/mL)-treated group showed a decrease in fluorescence intensity in the elongation phase, illustrating minimal Aβ fibrillation. In addition, SEMG (100 μg/mL)-treated groups showed increased lag phase of aggregation, revealing the fact that SEMG might have stabilized the native unfolded peptide, thereby intervening the aggregation of Aβ peptide. In a Phase I study, Aβ (25–35) peptide showed a five- and eight-fold increase in fluorescent intensity at 20 and 48 h, revealing the formation of protofibrils and oligomers from Aβ monomer. Upon co-treatment with SEMG, the fluorescent intensity reduced by four-fold (4.15 ± 0.019 a.u.) and ten-fold (2.25 ± 0.03 a.u.) after 20 and 48 h, illustrating the fact that SEMG efficiently attenuated the formation of oligomers from Aβ monomers (Figure 4B). Results were further substantiated by CLSM images, which showed enhanced green fluorescent intensity in the vehicle control, while SEMG- and galanthamine-treated groups showed a reduction in fluorescent intensity (Figure 4C), revealing the ability of SEMG to inhibit aggregation of Aβ fibrils.

**Figure 5:**
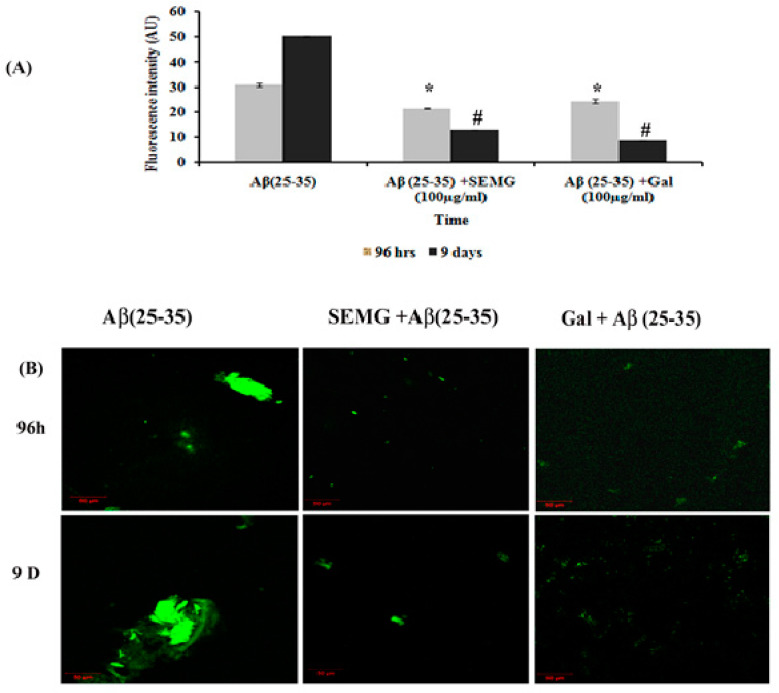
Effect of SEMG on the disintegration of preformed mature fibrils. (**A**) Th-T fluorescence assay, (**B**) confocal laser scanning microscopic analysis. *,# *p* < 0.05 represents statistically significant difference in comparison with treated groups with control and positive control group.

In a Phase II study, an increase in fluorescence intensity was observed in Aβ (25–35)-treated groups from 96 h (30.89 ± 0.63 a.u.) to 9 days (50.22 ± 0.24 a.u.), revealing the formation of mature plaques from oligomers. Treatment with SEMG and galanthamine showed a remarkable decline in fluorescent intensity by four- and six-fold when compared to vehicle control, indicating the disaggregation of mature fibrils and attenuation of self-assembly of mature fibrils to amyloid plaques by SEMG (Figure 5A). Furthermore, results of confocal microscopic studies showed enhanced green fluorescence intensity in the Aβ-treated group with a decline in fluorescent intensity in SEMG-treated groups (Figure 5B). The outcome of the study reveals that SEMG restored the anti-aggregation and fibril destabilizing ability of 7-MG, indicating that SEMG could be used as an anti-amyloidogenic agent for AD treatment.

##### SEMG Pretreatment Prevented Aβ-Induced Toxicity in Neuro2A Cell Lines

**Figure 6:**
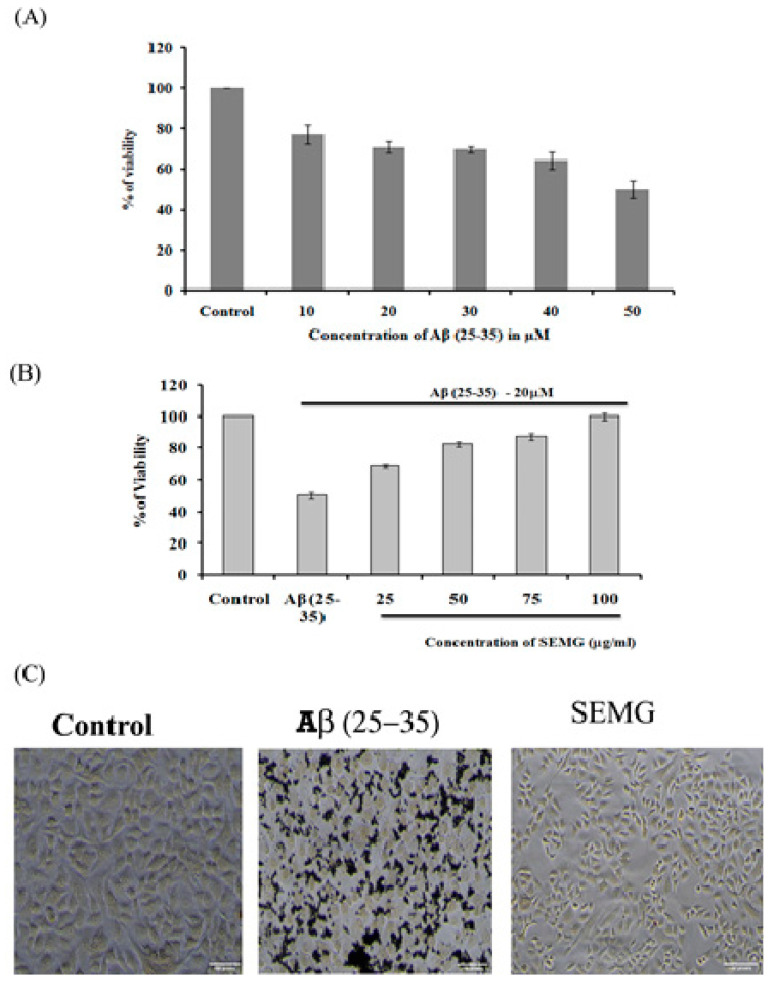
(**A**) Assessment of in vitro cytotoxic effect of Beta amyloid peptide (Aβ (25–35)) (10–500 μM) in Neuro2A cell lines; (**B**) Evaluation of protective effect of SEMG against Aβ (25–35) induced toxicity; (**C**) Phase contrast microscopic analysis to assess the morphological changes induced by Aβ (25–35) toxicity with magnification 20 ×.

The neuroprotective effect of SEMG against Aβ (25–35)-induced toxicity was assessed by the MTT assay. Results revealed that treatment of Neuro2A with Aβ (25–35) for 24 h reduced the viability of the cells dose-dependently, with a reduction by 49.72% ± 4.27% at the highest dose of 50 µM, and this dose was fixed for further studies (Figure 6A). Pretreatment with SEMG (25–100 µg/mL) resulted in the reduction of Aβ (25–35)-induced cytotoxicity dose-dependently, restoring the viability of the cells to 99.99% ± 1.98% at 100 µg/mL (Figure 6A). IC_50_ value of SEMG was observed to be 18.25 ± 0.025 µg/mL (Figure 6B). The increase in cell viability may be due to the high antioxidant potential of SEMG which could have attenuated the oxidative stress induced by Aβ (25–35) toxicity. Furthermore, the protective effect of SEMG against Aβ (25–35)-induced toxicity was further substantiated by trypan blue exclusion studies (Supplementary Figure S1). Phase contrast microscopic image of Aβ (25–35)-treated cells revealed the presence of classical epithelial morphology with tight cell-to-cell adhesion in vehicle control, while Aβ (25–35)-treated cells showed round and shrunk cells with loss of extensions associated with reduction in cell count. In some cells, membrane blebbing with condensed nuclei were observed, illustrating characteristic features of apoptosis [41]. However, SEMG-treated cells showed morphological features similar to vehicle control, revealing the protective effect of SEMG against Aβ (25–35)-induced toxicity (Figure 6C).

##### Role of SEMG in Attenuating ROS and RNS (Reactive Nitrogen Species) Levels Induced by Aβ (25–35) Toxicity

**Figure 7:**
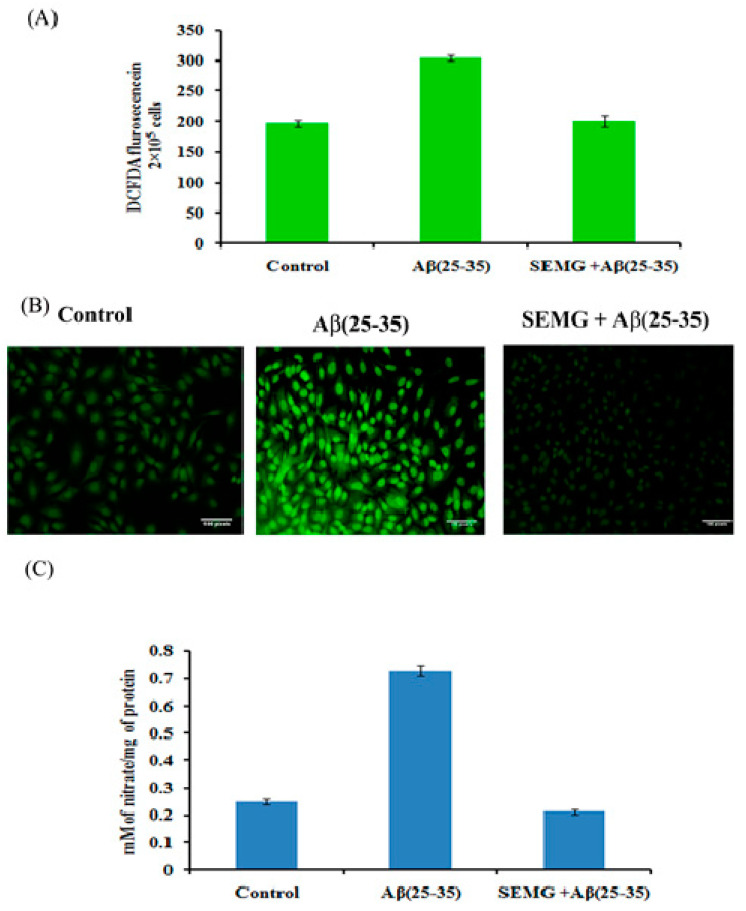
(**A**) Measurement of ROS level in cells exposed to Aβ (25–35) co-treated with SEMG by spectro fluorimetric analysis; (**B**) Fluorescence microscopic images of Neuro2A cells illustrating intracellular ROS level with 20 × magnification; (**C**) RNS scavenging effect of SEMG in cells treated with Aβ (25–35). Data are expressed as Mean ± SD of triplicate assays.

Multiple evidences revealed that Aβ peptide during oligomerization induced the release of H_2_O_2_ in the presence of transition metal ions Fe^2+^ or Cu^2+^, which in turn promotes Fenton chemistry enhancing the production of ROS and RNS, leading to lipid peroxidation and protein oxidation [42,43]. In the present study, the effect of SEMG was assessed by measuring intracellular ROS and RNS levels. DCFH-DA, a fluorescent probe, was used to assess the cellular ROS level. Results revealed that cells treated with Aβ showed a one-fold increase in fluorescent intensity (303.66 ± 4.92 a.u) when compared to vehicle control (196.33 ± 4.92 a.u.), while SEMG-treated groups attenuated Aβ-induced ROS level, restoring it to normal (Figure 7A). Confocal microscopic images showed increased green fluorescence in Aβ-treated groups, while SEMG-treated groups showed reduced green fluorescence similar to vehicle control (Figure 7B). 

Aβ (25–35)-treated cells showed a significant increase in nitric oxide level (0.729 ± 0.018 mM of nitrate/mg of protein), while SEMG-treated groups reduced the nitric oxide level (0.213 ± 0.008 mM of nitrate/mg of protein) similar to vehicle control (0.249 ± 0.019 mM of nitrate/mg of protein), which might be due to the antioxidative potential of SEMG (Figure 7C).

##### Effect of SEMG on Lipid Peroxidation and Protein Oxidation Induced by Aβ (25–35) Toxicity

**Figure 8:**
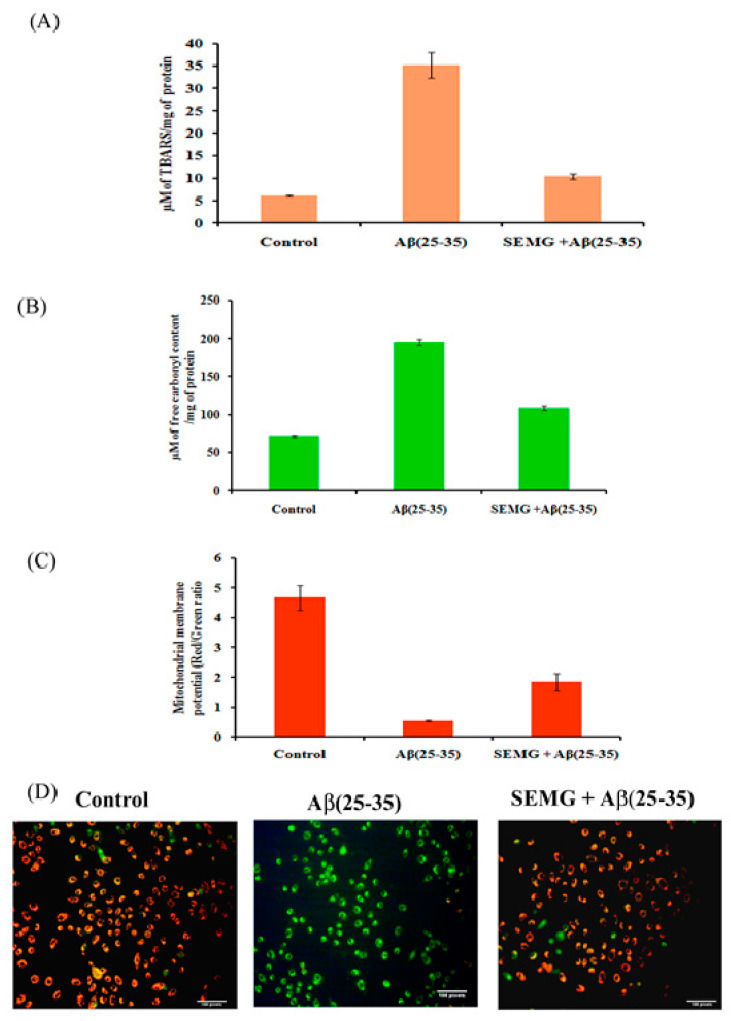
Effect of SEMG on Aβ(25–35) induced (**A**) lipid peroxiation; (**B**) protein oxidation in Neuro2A cells; (**C**) Bar diagram illustrating the restoration of MMP loss in cells treated with SEMGF; (**D**) Fluorescent microscopic analysis representing the prevention of loss of MMP by SEMG treated groups using JC-1 staining with 20 × magnification.

Multiple laboratory and clinical evidences revealed the presence of elevated levels of oxidative stress markers of lipid peroxidation and protein oxidation, like 4-hydroxynonenal and protein carbonyl content, in the brain of AD patients [44,45]. To verify the free radical scavenging activity of SEMG, the degree of lipid peroxidation and protein oxidation with thiobarbituric acid reactive substances (TBARS) and protein carbonyl content (PCC) as indicators was assessed in Aβ-exposed cells pretreated with SEMG. In the present study, cells treated with Aβ showed a five-fold increase in TBARS and a two-fold increase in PCC content (195 ± 4.47 µM of free carbonyl content/mg of protein), while SEMG-treated cells averted lipid peroxidation (35.13 ± 2.86 µM of TBARS/mg of protein) and protein oxidation (109 ± 3.22 µM of carbonyl content/mg of protein), restoring its level similar to vehicle control (Figure 8A, B). The restoration of TBARS and protein carbonyl content of SEMG might be due to the antioxidant potential of MG, which quenched the ROS and RNS liberated by Aβ-induced toxicity.

##### SEMG Protected Neuro2A Cells from Mitochondrial Membrane Potential (MMP) Loss Induced by Aβ Toxicity

The changes in MMP induced by Aβ toxicity were assessed by using lipophilic fluorescent dye JC-1. Fluorescent microscopic studies revealed that vehicle control cells showed uniformly red fluorescent cells (JC-1 aggregates), indicating the presence of intact mitochondrial transmembrane, while Aβ-treated cells showed loss of red fluorescent cells complemented with appearance of green fluorescence cells (JC-1 monomer), revealing the fact that dye diffused into cytoplasm due to disruption of MMP—the initial step of the apoptotic pathway (Figure 8C). However, SEMG-treated cells revealed the presence of red fluorescent cells, depicting the fact that SEMG attenuated ROS-induced disruption in MMP. Quantitative analysis illustrated that cells treated with Aβ (25–35) showed an eight-fold reduction in the ratio of red/green fluorescence intensity, revealing the fact that Aβ triggered MMP collapse (Figure 8D), while SEMG-treated cells restored the ratio (3.08 ± 0.51) similar to vehicle control (4.67 ± 0.424), depicting the fact that SEMG attenuated ROS-induced MMP loss, thereby blocking the activation of the apoptotic pathway.

##### SEMG Impedes Apoptosis in Neuro2A Cells Induced by Aβ Toxicity

**Figure 9:**
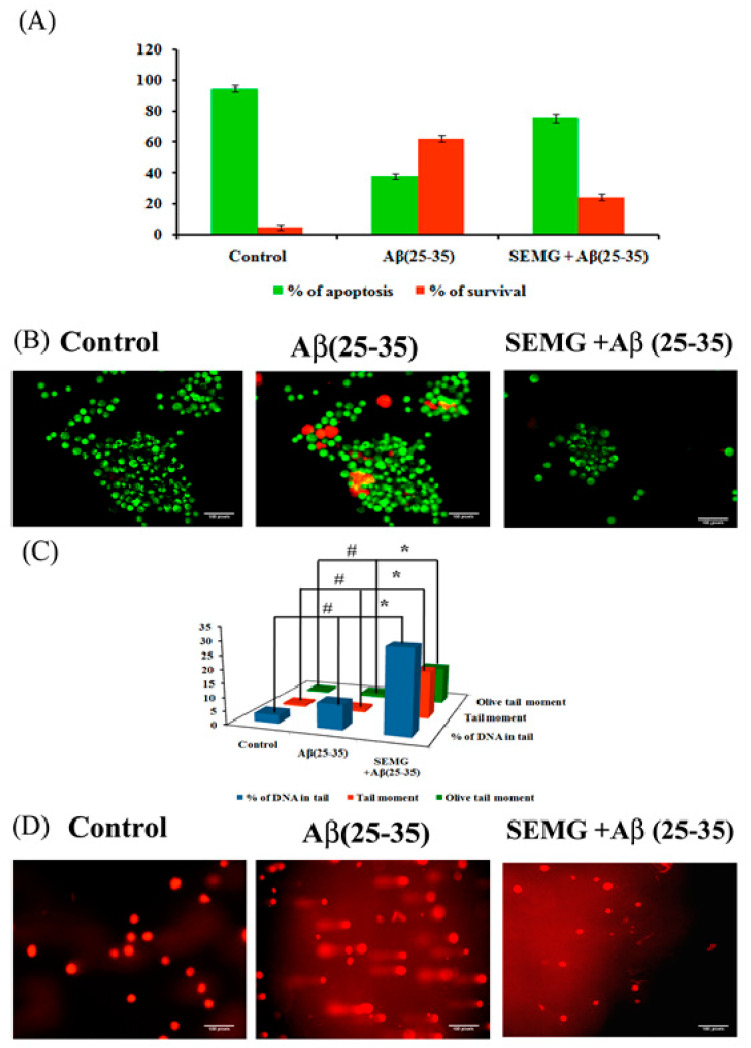
Assessment of antiapoptotic effect of SEMG in Aβ treated Neuro2A cells by Acridine orange/Ethidium Bromide dual staining technique (**A**) Quantification of apoptotic population; (**B**) Fluorescent microscopic image revealing the presence of viable cells, apoptotic and necrotic cells * & # *p* < 0.05 denotes the statistical significance between the control and treated groups with respect to viable and apoptotic cells; (**C**) Bar diagram illustrating the SEMG ability to attenuate the DNA damage in cells treated with Aβ(25–35); (**D**) Fluorescent microscopic images illustrating the DNA damaging effect of Aβ(25–35) treatment and attenuating effect of SEMG by comet assay at 20 × magnification. Data are expressed as mean ± SD of triplicate assays. Images were captured at 20 × magnification respectively.

Loss of MMP due to Aβ toxicity leads to activation of the apoptotic pathway, leading to neuronal death. In the present study, the anti-apoptotic effect of SEMG against Aβ-induced toxicity was assessed by the AO/EtBr dual-staining technique, widely used to identify morphological changes associated with apoptosis. Results showed the presence of uniformly stained green fluorescence cells, illustrating viable cells in vehicle control, while Aβ-treated cells showed the presence of orange red fluorescent cells, indicating late apoptotic stage (Figure 9B). SEMG co-treated cells showed the presence of green fluorescence cells, indicating the fact that MG attenuates Aβ-induced apoptosis. Quantification studies revealed the presence of increased percentage of apoptotic cells in Aβ (25–35)-induced toxicity (62.33% ± 1.86% and 37.66% ± 1.9% apoptotic and viable cells) when compared to vehicle control (95% ± 1.78% and 5% ± 1.8% live and apoptotic cells), while SEMG-treated cells showed a reduction in apoptotic cells (24.66% ± 2.25%) and in increase in viable cell count (75.33% ± 2.3%) (Figure 9A). The results illustrated that SEMG exhibited an anti-apoptotic effect.

##### Protective Effect of SEMG Against Aβ-Induced Genotoxicity

To assess the genotoxic effect of Aβ (25–35) in Neuro2A cells, single-cell gel electrophoresis was carried out. Fluorescence microscopic images showed the presence of large non-fragmented intact nucleoids in vehicle control-treated cells, while Aβ-treated cells showed an increase in the length of DNA tail (DNA damage), which provided the appearance of comet during alkaline gel electrophoresis (Figure 9C). SEMG-treated cells showed intact cell morphology similar to vehicle control. Figure 9D illustrates the levels of DNA damage in terms of % of tail DNA, length of the tail and tail moment. Vehicle control cells showed 97% ± 0.09% head DNA with 3.09% ± 0.02% tail DNA respectively, while Aβ-treated groups showed 30.33% ± 0.003% of DNA in tail, depicting the fact that Aβ induced apoptotic cell death (Figure 7B). SEMG-treated cells exhibited 91.09% ± 0.02% of head DNA and 9.02% ± 0.009% of DNA in tail, depicting that MG in SEMG attenuated ROS-mediated DNA damage induced by Aβ toxicity.

## 4. Conclusions

The present study reported, for the first time, fabrication of starch-encapsulated methyl gallate by the graft polymerization technique. Fabricated SEMG showed high encapsulation efficiency and sustained release of drug MG under physiological pH 7.4. SEMG exhibited acetylcholinesterase inhibitory activity and antioxidant capacity. In addition, SEMG also attenuated aggregation of Aβ peptide and disaggregated the preformed amyloid plaques, illustrating the fact that SEMG restored the anti-amyloidogenic effect of MG. SEMG exhibited neuroprotective effect by attenuating ROS-mediated mitochondrial dysfunction and DNA damage induced by Aβ toxicity in Neuro2A cells. To conclude, the results of the present study revealed that starch-based nano-carriers are suitable for the delivery of the drug methyl gallate for the treatment of Alzheimer’s disease and other related neurodegenerative disorders.

## Supplementary Materials

The following are available online at https://www.mdpi.com/1999-4923/13/3/299/s1, Figure S1: (A) Assessment of in vitro cytotoxic effect of Aβ (25–35) (10–50 M) in Neuro 2A cell lines; (B) Evaluation of protective effect of SEMG against Aβ (25–35) induced toxicity by trypan blue exclusion assay.
