# Supplementary material for: Decoding the Neuroprotective Potential of Methyl Gallate-Loaded Starch Nanoparticles against Beta Amyloid-Induced Oxidative Stress-Mediated Apoptosis: An In Vitro Study
Source: Pharmaceutics. 2021 Feb 25;13(3):299. doi: 10.3390/pharmaceutics13030299 (PMC7996348; doi:10.3390/pharmaceutics13030299)
Supplement: Supplementary file 1 [file pharmaceutics-13-00299-s001.zip › pharmaceutics-1100974-supplementary.pdf]

pharmaceutics

MDPI

Supplementary Materials: Decoding the Neuroprotective Potential of Methyl Gallate-Loaded Starch Nanoparticles against Beta Amyloid-Induced Oxidative Stress-Mediated Apoptosis: An In Vitro Study

Prakashkumar Nallasamy, Bhagavathi Sundaram Sivamaruthi, Chaiyavat Chaiyasut and Natarajan Suganthy

Trypan blue exclusion assay:

Methdology

Cytotoxic effect of Aβ (25-35) in Neuro2A cells were evaluated by trypan blue dye exclusion method, Neuro2A (1×10^6 cells) were treated with various dose of Aβ (25–35) (10 –50 µM) for 24 h at 37 °C to fix the lethal dose. After the toxic dose fixation, the cells were pretreated with different concentration of SEMG (20-100 µg/ml) followed by exposure to Aβ (25–35) at the toxic dose of 20 µM for 24 h at 37 °C. Following treatment, the cells were subjected to PBS wash and incubated with trypan blue (10 µg/mL) for 10 min. Number of live (transparent) and dead cells (blue) were analyzed using hemocytometer and the percentage of live cells was calculated.

Results

Figure S1 showed dose dependent decrease in the cell viability with 71.23 ± 0.02% viability at 20 µM concentration of Aβ (25–35) correlating the results of MTT and this dose was fixed as toxic dose for neuroprotective studies of SEMG. Exposure to 20 µM of Aβ (25–35) showed decrease in cell viability by 65.23 ± 0.023% while cells, pretreated with SEMG restored the viability of cells exposed to Aβ (25–35) in dose dependant manner exhibiting 92.25 ± 0.03% viable cells at 100 µg/ml of SEMG revealing the neuroprotective effect of SEMG against Aβ toxicity.

(A)

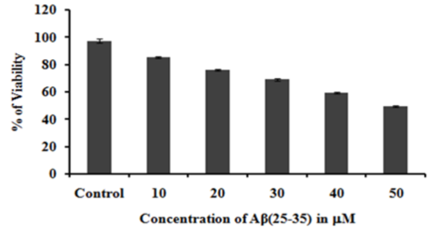

(B)

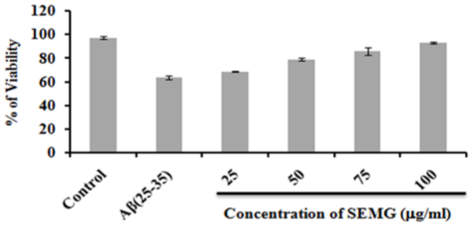

Figure S1: (A) Assessment of *in vitro* cytotoxic effect of Aβ (25–35) (10–50 ◎M) in Neuro 2A cell lines; (B) Evaluation of protective effect of SEMG against Aβ (25–35) induced toxicity by trypan blue exclusion assay.
